# Multiple magnetic transition and magnetocaloric properties in the mixed valence Eu_8_CuNi_2.5_Si_42.5_ type I clathrate compound

**DOI:** 10.1016/j.heliyon.2024.e30381

**Published:** 2024-04-26

**Authors:** Pooja Rawat, Seung Hun Cha, Jin Hee Kim, Jae Hyun Yun, Jong-Soo Rhyee

**Affiliations:** a Department of Applied Physics, Integrated Education Institute for Frontier Science and Technology (BK21 Four) and Institute of Natural Sciences, Kyung Hee University, Yongin, 17104, South Korea; b Department of Chemistry, Biochemistry and Forensic Science, Amity School of Applied Sciences, Amity University Haryana, Gurugram, Haryana, 122413, India

**Keywords:** Magnetocaloric, Mixed valence, Ferromagnetic, Type I clathrate, Antiferromagnetic

## Abstract

We investigated the magnetocaloric and electrical transport properties of the Eu_8_CuNi_2.5_Si_42.5_ clathrate compound, synthesized by an arc melting and annealing method. X-ray photoemission spectroscopy revealed a mixed valence state of Eu^2+^ and Eu^3+^. The low-field and low-temperature magnetic measurements indicated a multiple magnetic transition, from ferromagnetic near 35 K to antiferromagnetic at 25 K. Increasing the magnetic field led to the broadening of antiferromagnetic peaks and a final ferromagnetic state under high magnetic fields, indicative of spin reorientation. The transition from a ferromagnetic to an antiferromagnetic state was further corroborated by specific heat measurements. We noted spontaneous magnetization at low temperatures via magnetic hysteresis and Arrott plot analysis. The coexistence of an antiferromagnetic ground state (attributed to the Eu^2+^ ions) and ferromagnetic clusters (associated with the Ni^2+^ ions) was supported by spontaneous magnetization at low temperatures in the antiferromagnetic state. The magnetocaloric analyses revealed a high spin entropy change over a broad temperature range for Eu_8_CuNi_2.5_Si_42.5_, which implies its potential as a robust low-temperature magnetocaloric material, distinguished by its high refrigerant capacity.

## 1 Introduction

Type-I intermetallic clathrates have a cage structure that encloses and traps guest ions. The guest ions are weakly bound and encapsulated by a covalently bonded framework of host atoms. The physical properties of clathrates endow them with surprising functionalities, including thermoelectricity, hydrogen storage, magnetism, and superconductivity, all of which are strongly influenced by their guest atom elements [[1], [2], [3], [4], [5], [6]]. The host cage structure of type-I clathrates forms a sp^3^ hybridization with elements such as Si, Ge, and Sn host atoms. There have been extensive studies on the magnetic and thermoelectric applications of Si-based type-I clathrates [[7], [8], [9], [10]]. Although the clathrates are composed of metals, the motion of the guest ions within their cages scatters phonons significantly, resulting in low lattice thermal conductivity. In other words, by reducing heat transmission through the lattice, the phonon scattering makes these materials of interest for thermoelectric applications.

The magnetocaloric effect (MCE) is a thermomagnetic phenomenon wherein a material's temperature experiences alterations upon exposure to fluctuating magnetic fields [[11], [12], [13]]. Magnetic entropy (Sm) and its differential play pivotal roles in quantifying the MCE and the magnetic contribution to heat capacity. The overall entropy of a magnetic material, maintained at constant pressure, can be generally expressed as:

$$S_{T}\;\left(H,T\right)=S_{m}\;\left(H,T\right)+S_{r}\;\left(T\right)+S_{e}\;\left(T\right)$$

iin this equation, the composite entropy S_T_ comprises the magnetic entropy associated with the material's magnetization (Sm), the lattice entropy arising from vibrational motions of the crystal lattice (Sr), and the electronic entropy originating from the free electrons in the material (Se) [13]. Notably, the lattice and electronic entropies remain largely unaffected by variations in the magnetic field, responding solely to temperature fluctuations. In contrast, magnetic entropy is markedly responsive to changes in both the magnetic field and temperature. The magnetic entropy, however, is highly dependent on both the magnetic field and the temperature. When a magnetic material is exposed to a magnetic field, the spins of the atoms or ions tend to align with the field direction, leading to a decrease in magnetic entropy. This alignment contributes to an increase in the ordering of magnetic moments, which is related to the decrease in magnetic entropy. Simultaneously, the phase transitions or changes in lattice structure that may occur as a result of the applied magnetic field can lead to changes in lattice entropy and thus contribute to a change in temperature. In summary, the magneto caloric effect is a manifestation of changes in both spin entropy and lattice entropy in response to an external magnetic field. When the alignment of magnetic moments is favored, it leads to a decrease in magnetic entropy and an increase in temperature. This is a highly relevant and useful phenomenon in the field of magnetic refrigeration, as it allows for efficient and environmentally friendly cooling systems.

When the magnetic field is removed, the material's magnetic moments become disordered again, absorbing heat and causing the material to cool down. MC refrigeration is type of a solid-state cooling that exploits this property, and so does not use environmentally harmful gases. MCE cooling performance is directly related to the high spin entropy change [[14], [15], [16]].

Akai et al. and Koga et al. found that substituting rare-earth elements (such as Eu and Yb) for Ba atoms in the crystal structure alters the conduction band edges at the X point. This modification enhances the effective mass of carriers, affecting how electrons move through the material and its electrical properties [17,18]. Rare-earth-based type-I clathrates exhibit intriguing magnetic properties such as ferromagnetism, antiferromagnetism, spin glass, frustrated magnetism, mixed valent, and heavy fermion properties, because of their rare-earth element guest atoms. These features stem from their partially filled 4f electron shells [19,20]. The rattling of the rare-earth guest atoms can also lead to a high magnetic entropy change, which can be exploited for magnetocaloric refrigeration [8]. We propose that rare-earth-based clathrates are good candidates for high spin entropy change because of their local movement in the clathrate cages, and the weak interaction of magnetic ions. For example, a Eu-based clathrate has shown high MCE properties at cryogenic low temperatures [8].

Here, we investigate the magnetic ground state and magnetocaloric properties of the clathrate compound Eu_8_Cu_1.0_Ni_2.5_Si_42.5_. We found a multiple magnetic ordering, from a ferromagnetic (T_c_ = 35 K) to antiferromagnetic state (T_N_ = 25 K), in a low magnetic field with decreasing temperatures. X-ray photoelectron spectroscopy (XPS) measurements indicated, there was a mixed valance state of europium (Eu^2+^/Eu^3+^). Isothermal magnetization measurements and magnetocaloric analysis revealed a moderately large entropy change 2.6 J kg^−1^ K^−1^ (T = 31 K and H = 5 T) over a wide temperature range, resulting in a large refrigerant capacity. This high spin entropy change is comparable to those of previously reported Eu-based intermetallic MCE materials [[14], [15], [16]]. This research suggests that rare-earth-based clathrate compounds are good candidates for unconventional magnetism, and highly efficient MCE materials at cryogenic low-temperature applications.

## 2 Experimental details

### 2.1 Materials synthesis

Eu_8_CuNi_2.5_Si_42.5_ clathrate was prepared by arc melting method using high-purity metallic elements. The high-purity elements of Eu (Alfa Aesar, 99.9 %) Ni (ITASCO, 99.9 %), Cu (Alfa Aesar, 99.99 %), and Si (R&D Korea, 99.99 %) were weighed (total mass 5 g) in an argon-filled glove box according to the stoichiometry Eu_8_CuNi_2.5_Si_42.5_.10 % excess Eu was added to overcome losses during arc melting. For homogeneity, the melting process was repeated 4–5 times by flipping. The arc-melted ingot was grounded into a fine powder using an agate pestle and mortar. The minor impurities such as EuSi_2_, Si were removed by acid-base treatment (stirring with 3 M HCl for 8 h and after that 1 M NaOH for 8 h). Then the washed powder sample was pressed into a pellet using a cold press and sealed in an evacuated quartz tube for vacuum annealing at 800 °C for 48 h. The density of the annealed pellet was calculated to be 3.36 g cm^−3^.

### 2.2 Characterizations

The structural, morphological, and chemical properties of the Eu_8_CuNi_2.5_Si_42.5_ clathrate were studied using X-ray diffraction (XRD), Raman spectroscopy, scanning electron microscopy, energy dispersive X-ray spectroscopy (SEM-EDX), and X-ray photoelectron spectroscopy (XPS), respectively. The crystal structure was characterized using a D8 advanced diffractometer with Cu K_α_ radiation (Rigaku, Japan). The Raman spectra were measured using Renishaw's in Via Raman microscopes. The morphology and composition analysis was carried out by SEM (MERLIN, Carl Zeiss, Germany) and EDX (Oxford instrument, England). The oxidation state of the Europium was also measured by XPS analysis using K-Alpha (Thermo Fisher Scientific, U.S.A.). The magnetization was measured using a physical property measurement system (PPMS Dynacool 14T, Quantum design, U.S.A.) with a vibrating sample magnetometer (VSM) option. The electrical resistivity was measured by 4-point probe contact method using a cryogen-free physical property measurement system (PPMS Dynacool 14T, Quantum design, U.S.A.) under a magnetic field ranging from 0 to 8T. The heat capacity was also measured by PPMS using the temperature relaxation method.

## 3 Results and discussion

### 3.1 Structural analysis

**Fig. 1:**
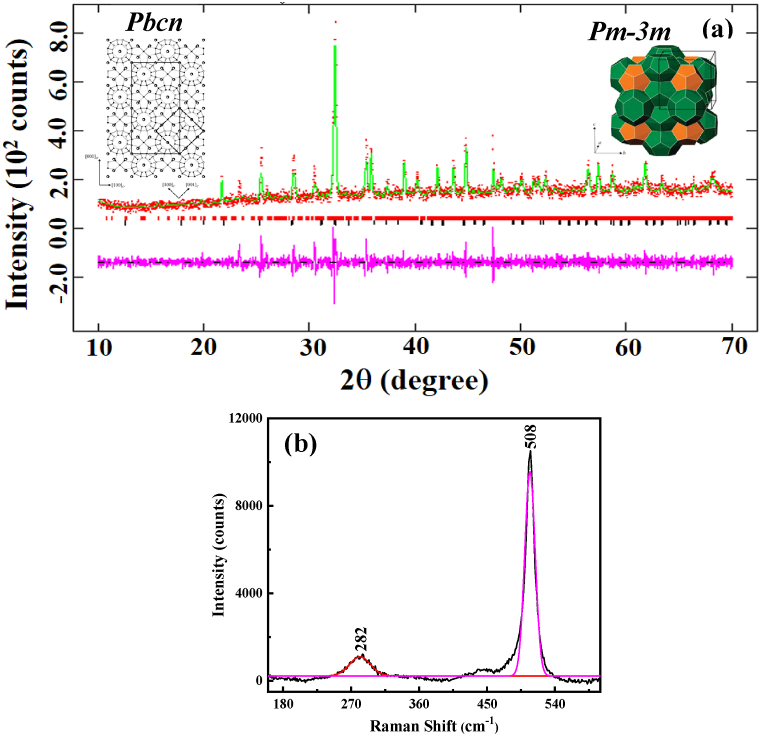
(a) Structure refinement of the Eu_8_CuNi_2.5_Si_42.5_ clathrate using two phases. The Bragg positions correspond to cubic (*Pm-3n*; black color), and orthorhombic (*Pbcn1*, red color) structures. The red color pattern shows the experimental data while the green color plot represents the fitted data. The pink color line shows the extent of fitting between the experimental and the fitted pattern, (b) Raman spectrum of the Eu_8_CuNi_2.5_Si_42.5_ clathrate. The black color pattern is the experimental data, the pink dotted color curve is the fitting lines for the high intensity peak (508 cm^−1^), and the red color pattern is the fitted curve for the broad hump (282 cm^−1^). The Type I clathrate structure is shown in the inset of Fig. 1(a) [22]. (For interpretation of the references to color in this figure legend, the reader is referred to the Web version of this article.)

Fig. 1(a) shows the X-ray diffraction pattern with crystal structure refinement. Indexing of the powder diffraction pattern showed the presence of two phases; one, a major phase, was a cubic (*Pm-3m*) type I clathrate, while the other was the orthorhombic (*Pbcn*) superstructure. The ionic radii of Eu^2+^ was 131 p.m., while Ba^2+^ was 149 p.m. Since both ions have similar ionic radii, europium can easily occupy the Ba^2+^ cage sites. Depending on the synthetic condition, the presence of minor secondary phases is commonly observed in type I clathrates [[8], [9], [10]]. The minor orthorhombic structure is not an extrinsic phase separation but a low crystalline symmetry due to thermodynamic stability [10].

The structure refinement was performed using GSAS software, and two different phase parameters [21,22]. The substitution of the transition metal in the host position or a change in guest elements causes a change in symmetry, from cubic to orthorhombic, as discussed by Kovnir et al. [21]. In the Eu_x_Ba_1-x_CuP_30_ clathrate, instead of a cubic structure, the composition changed to a larger unit cell (orthorhombic symmetry). The phase transition in Eu_8_Ga_16_Ge_30_ from a low temperature α (type VII clathrate) to a high-temperature β (type I clathrate) was also discussed by Stefano et al. [23].

The calculated lattice parameters after the structure refinements using two phases were *a* = 9.9471 (14) Å for the *Pm-3m* (cubic structure) and *a* = 14.083 (14) Å, *b* = 10.0559 (8) Å and *c* = 27.95 (27) Å, for the *Pbcn* (orthorhombic) space group. This group-subgroup relation can be described as a combination as “kassengleich” (class equivalent) and “translationengleich” (lattice equivalent) symmetry reductions [21]. All of the crystallographic parameters are listed in Table 1.

**Table 1:** Crystallographic parameters of the Eu_8_CuNi_2.5_Si_42.5_ clathrate obtained after the structure refinement using GSAS software.

Compound	Eu_8_CuNi_2.5_Si_42.5_	
Structure	Cubic	Orthorhombic
Space group	*Pm-3n*	*Pbcn*
*a* (Å)	9.9471 (14)	14.083 (14)
*b* (Å)	9.9471 (14)	10.0559 (8)
*c* (Å)	9.9471 (14)	27.95 (27)
*V* (Å^3^)	984.2 (4)	3958 (4)
*Z*	4	
density (gcm^−3^)	3.36	
2θ range	10–70	
R_p_ (%)	0.1032	
R_wp_ (%)	0.0781	
*χ*^2^	1.589	

Inorganic clathrates share similar structures, including a polyhedral cage with 20 or 28 vertices and guest ions encapsulated within those vertices. The ideal structure of a type I clathrate (Ba_8_Si_46_) is cubic *Pm-3n* (223), where the host is expected to occupy 6c, 16i, and 24 k and the guest sits inside the host cage (Fig. 1. (a) inset) [22].

Raman spectroscopy was used to characterize the vibrational modes of the Eu_8_CuNi_2.5_Si_42.5_ clathrates, as presented in Fig. 1(b). The two peaks of the Raman spectrum of silicon clathrates indicate different phonon modes: 1) The peak in the high-frequency framework signifies the robust interaction between the guest atoms and the framework; 2) the low-frequency vibration peak is related to the rattling of guest atoms [24]. Generally, in non-substituted clathrates a high-frequency peak for the host framework is observed between 430 and 461 cm^−1^, e.g. at 438 cm^−1^ in Ba_8_Si_46_ [25]. When there is a substitution of other elements, red/blue shifts in peak position occur because of the change in bond length [24]. A red shift was also observed in the lower wavenumber peak, between 100 cm^−1^ to 200 cm^−1^ in the non-substituted, and at 250-300 cm^−1^ in the substituted silicon clathrate framework. For the Eu_8_CuNi_2.5_Si_42.5_ clathrate, the peak position of the sharp band was at 508 cm^−1^, while a broad band was observed at 282 cm^−1^, as shown in Fig. 1 (b).

### 3.2 Microstructural and chemical analyses

**Fig. 2:**
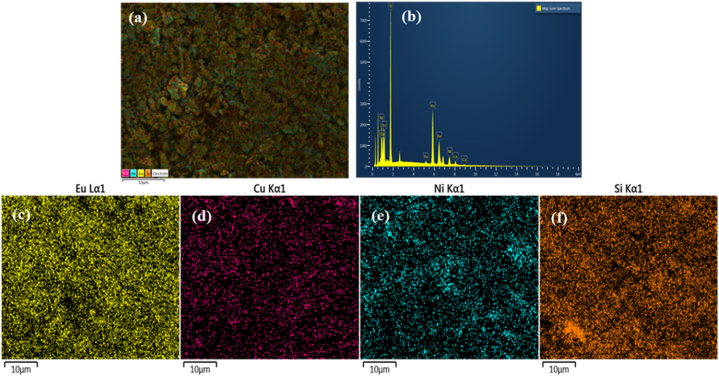
(a) SEM with (b) EDX analysis of Eu_8_CuNi_2.5_Si_42.5_ as seen in the backscattered electron microscopic image, with elemental mapping across the whole field of the polished surface for the Eu (c), Cu (d), Ni (e), and Si (f) elements, respectively.

Fig. 2(a) and (b) represent the scanning electron microscopy (SEM) image with elemental mapping and the energy dispersive X-ray spectroscopy (EDX) measurements. The elemental mappings of Eu, Cu, Ni, and Si are shown in Fig. 2(c)∼(f), respectively. Depending on the type of minority phase, the chemical composition of these specific elements varies, and they are randomly dispersed within the clathrate phase matrix. The elemental distribution map shows an equal distribution of different elements with a slight excess of Eu elements. From the quantitative EDS analysis in Fig. 2(b), the elemental concentrations in the Eu clathrate composition were calculated to be Eu_14.29_Cu_1.95_Ni_2.06_Si_37.58_. The presence of extra Eu in the EDS analysis may be due to the extra Eu added during the reaction to compensate losses during the arc melting synthesis.

**Fig. 3:**
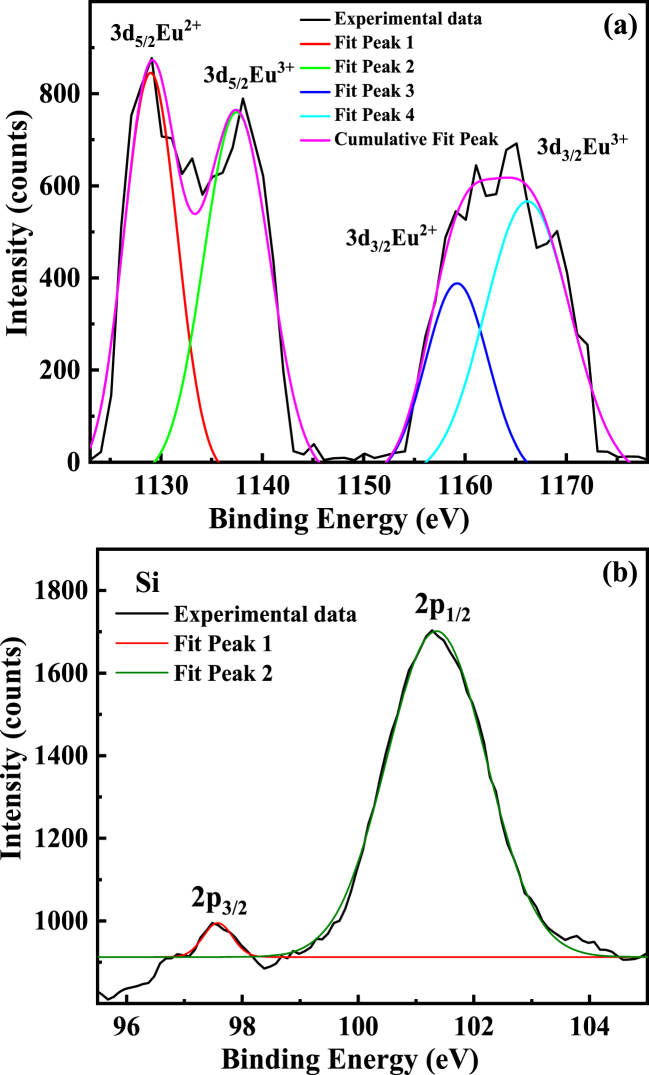
High-resolution XPS spectra of the Eu_8_CuNi_2.5_Si_42.5_ clathrate near binding energies for mixed valence Eu (a) and Si peaks (b). The black line is the experimental data and the colored lines are the fitted peaks, as indicated.

To understand the chemical surface state of the clathrates, we used soft x-ray photoelectron spectroscopy (XPS). The XPS spectra near the binding energies of the Eu 3d and Si 2p are presented in Fig. 3. Valance band resonance experiments are helpful to check the type of orbitals involved in the valance band formation. Eu 3d peaks were observed between 1100 and 1200 eV [26]. The Si 2p peak was observed at 98 (2P_3/2_) and 101 eV (2P_1/2_), corresponding to a Si clathrate framework [26].

The average Eu valance can be estimated by the deconvolution of the Eu L_3_ edge spectrum fitted with Gaussian–Lorentzian functions after background subtraction. The deconvoluted Eu core level XPS spectrum showed that Eu exists in the mixed valance state of Eu^2+^ and Eu^3+^, as shown in Fig. 3. The region from 1120 to 1145 eV belongs to 3d_5/2_ for both Eu^2+^-Eu^3+^ and the region from 1150 to 1175 eV belongs to 3d_3/2_ for Eu^2+^-Eu^3+^, respectively [26].

The relative concentration of the Eu^2+^/Eu^3+^ions was calculated using the area under the deconvoluted peak from Fig. 3(a). The calculated area under the curve comes to be 43 % for Eu^2+^ and 57 % for Eu^3+^, respectively. The change in the oxidation state can be explained by the gradual lattice contraction, which favors the presence of the Eu^3+^ state in the Eu_8_CuNi_2.5_Si_42.5_ clathrate sample. Fig. 3(b) shows the X-ray photoelectron spectroscopy (XPS) peaks of Si 2p orbitals. The Si 2p_1/2_ peak is more pronounced rather than the 2p_3/2_, indicating the localized bonding with lower symmetry of Si.

### 3.3 Magnetic properties

**Fig. 4:**
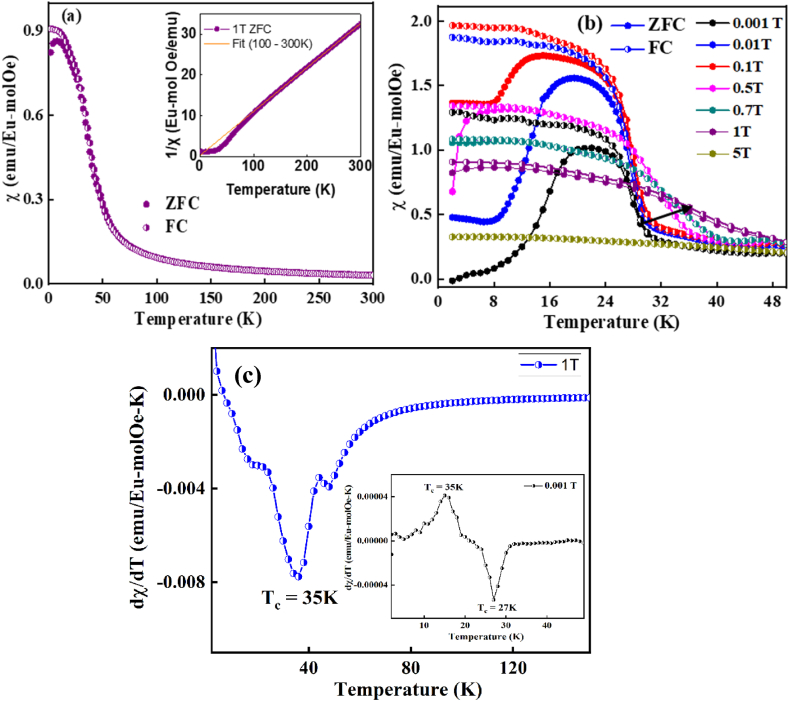
Temperature-dependent magnetic susceptibility χ(T) of the Eu_8_CuNi_2.5_Si_42.5_ clathrate. (a) Zero-field-cool (ZFC, closed symbol) and field-cooled (FC, half closed symbol) for H = 1 T. The inset shows the temperature-dependent inverse magnetic susceptibility 1/χ(T) and Curie-Weiss fit in the high-temperature region, (b) ZFC (closed symbol) and FC (half closed symbol) thermal hysteresis at different magnetic fields, as indicated.

The temperature-dependent magnetic susceptibility χ(T) of the Eu_8_CuNi_2.5_Si_42.5_ clathrate was measured from 2 to 300 K under static magnetic fields. Fig. 4(a) presents the zero-field-cooled and field-cooled χ(T) measurements under H = 1 T. A clear paramagnetic character was observed at high temperatures. The inset in Fig. 4(a) presents the temperature-dependent inverse magnetic susceptibility 1/χ(T), which follows the Curie-Weiss law at high temperatures (T ≥ 100 K). From the CW fit, the Weiss temperature was estimated to be Θ = -2.31 K, indicating weak antiferromagnetic interaction. The effective magnetic moment was calculated to be 8.6 μ_B_. The effective magnetic moments of Eu^2+^ and Ni^2+^ are 7.94 μ_B_ and 2.9–3.9 μ_B_, respectively. From the XPS analysis, we found that there was a mixed valence of Eu^2+^ and Eu^3+^ with a portion of 43 % and 57 %, respectively. The larger effective magnetic moment than those of Eu^2+^ (7.94 μ_B_) despite the existence of nonmagnetic Eu^3+^ is due to the additional contribution of Ni^2+^ (∼3.9 μ_B_).

Low field magnetic susceptibility measurements (H ≤ 0.5 T) showed there was significant thermal hysteresis between the zero-field-cooled (ZFC) and field-cooled (FC) measurements, as presented in Fig. 4(b). It is noteworthy that there are two magnetic transitions; one ferromagnetic (FM) transitions, at T_c_ = 35 K for H ≤ 0.5 T magnetic field, following an antiferromagnetic (AFM) transition (T_N_) or ferrimagnetic transition below 25 K at zero field cooling. To clarify the ferrimagnetic and antiferromagnetic transition below 25 K, it needs further investigation such as the neutron scattering or resonant X-ray scattering. The ferromagnetic transition temperatures were not sensitive to the applied magnetic fields, while the antiferromagnetic transition temperatures decreased with increasing magnetic fields in the ZFC measurement. To clearly understand the two transitions, a derivation magnetization was also calculated and plotted with respect to temperature. The derivative ZFC magnetization plot for higher magnetic field (1T) and lower magnetic field (0.001T) is presented as Fig. 4(c) and inset of Fig. 4(c), respectively. The two peaks at 27 K for FM and 17 K for AFM were observed at derivative magnetization plot for Eu_8.0_Cu_1.0_Ni_2.5_Si_42.5_ clathrate sample calculated for 0.001T zero field measurement.

Under a magnetic field higher than 0.5 T, the AFM maximum peak vanished, and the χ(T) only increased with decreasing temperature. A similar behavior relating to the multiple magnetic state between AFM and FM was also reported for EuPd_2_Sn_4_, EuPdSn_2_, and *R*Co_2_*Pn*_2_ (*R* = Rare-earth, Pn = P, As) compounds [[27], [28], [29]]. The FM to AFM multiple magnetic transitions with decreasing temperature were also observed in La_1-x_Pr_x_Co_2_P_2_ compounds [29]. From the theoretical calculation, it was suggested that the multiple magnetic states originated from the competing order of localized rare-earth and itinerant transition metal ions. The transition metal Co spins aligned within the ab-plane below the FM transition T_c1_. With further decreasing temperature, the rare-earth ions ferromagnetically aligned along the c-axis. At low temperatures, the ferromagnetic clusters of rare-earth ions and transition metal Co ions antiferromagnetically aligned, resulting in the antiferromagnetic transition.

The crystal structures in the cubic and orthorhombic type-I clathrate Eu_8_CuNi_2.5_Si_42.5_ compound can have similar behavior because a competing magnetic order can be expected between localized Eu ions and itinerant transition metal Ni ions. Previous investigations of the homologous compound EuCo_2_As_2_ also showed mixed valence Eu^2+^ and Eu^3+^ [29]. The multiple magnetic order originating from the competing order of rare-earth and transition metal ions should be investigated in further research, such as neutron scattering or resonant X-ray scattering experiments.

**Fig. 5:**
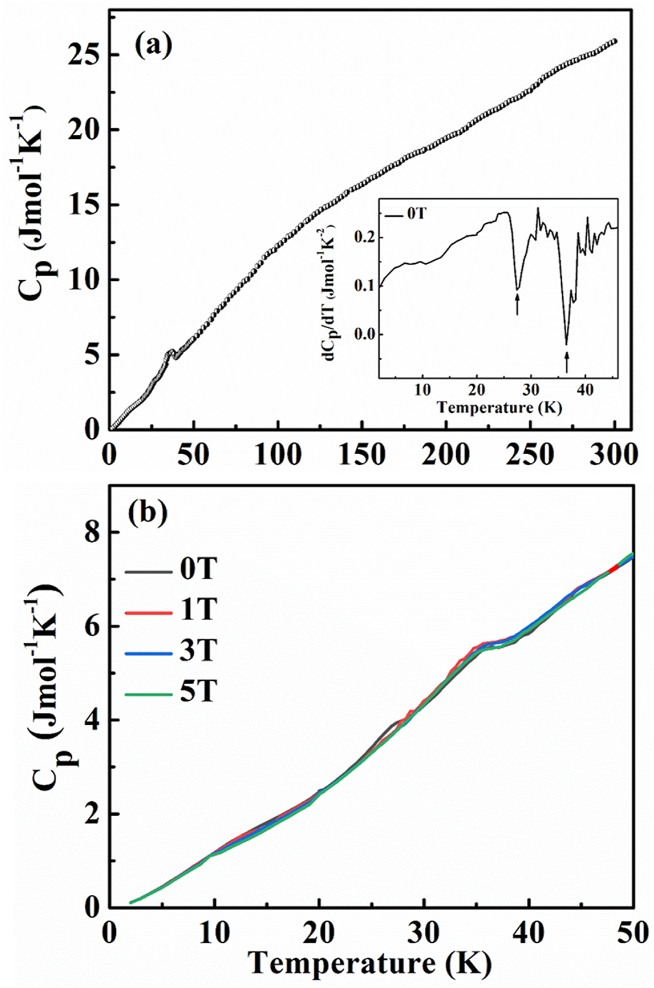
(a) Temperature-dependent specific heat C_p_(T) for 2 K ≤ T ≤ 300 K and temperature-derivative of specific heat dC_p_(T)/dT below T ≤ 50 K (inset) of Eu_8_CuNi_2.5_Si_42.5_ clathrate. (b) Temperature-dependent specific heat C_p_(T) under static magnetic fields of H = 0, 1, 3, 5 T for T ≤ 50 K.

The multiple magnetic transition is also clearly seen in the temperature-dependent specific heat C_p_(T) of the Eu_8_CuNi_2.5_Si_42.5_ clathrate, as shown in Fig. 5. In the low-temperature region, there are two signatures at 35 K and 24 K, marked by arrows, from the temperature derivative of specific heat dC_p_(T)/dT in the inset of Fig. 5(a). This may be associated with the magnetic ordering temperatures of FM and AFM orderings, respectively. Even though the magnetic ordering temperatures of C_p_(T) are a little bit higher than those from the χ(T), inconsistency in the ordering temperatures was widely observed. Small signatures at 24 K imply a weak AFM ordering, which leads to spin orientation in low magnetic field. The specific heat was not sensitive to the applied magnetic fields of H ≤ 5 T, as depicted in Fig. 5(b).

**Fig. 6:**
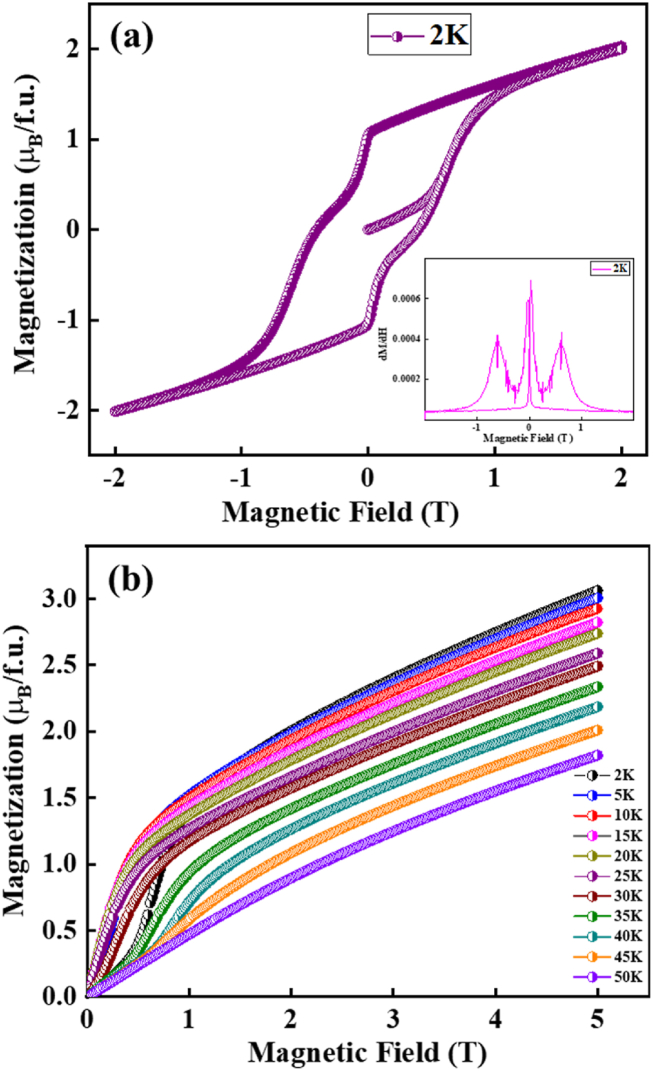
Isothermal magnetization with field for the Eu_8_CuNi_2.5_Si_42.5_ clathrate compound: (a) Isothermal magnetic hysteresis at T = 2 K. Inset is the magnetic field derivative of magnetization dM/dH at T = 2 K. (b) M − H curve at isothermal temperatures, as indicated.

The antiferromagnetic transition at low temperatures easily evolves to ferromagnetic spin alignment from the magnetic hysteresis, as presented in Fig. 6. In the low magnetic field region (H ≤ 0.7 T), the magnetization linearly increases, consistent with the AFM state, as shown in Fig. 6(b). Near H = 0.6 T, there is a spin orientation from the AFM to the FM state, which is clearly observed in the magnetization derivative with magnetic field dM/dH in the inset in Fig. 6(a). After the spin reorientation, the magnetic hysteresis behaved like ferromagnetic spin alignment [30].

**Fig. 7:**
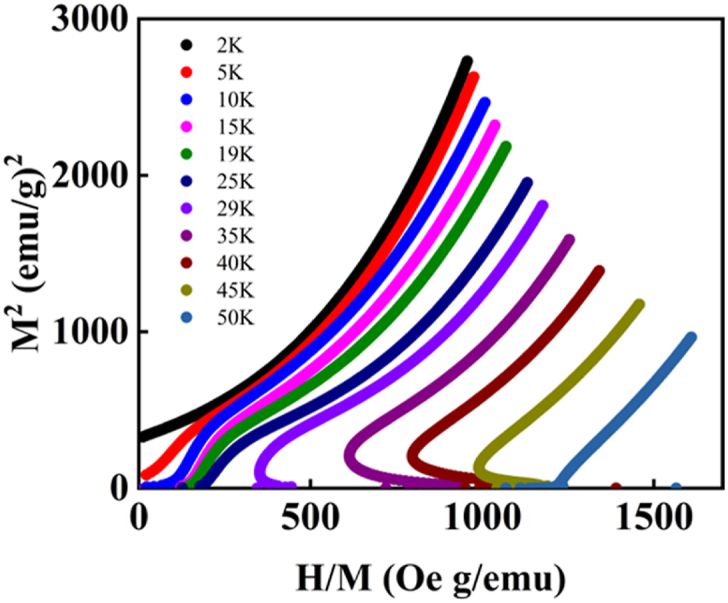
Arrot plot (M^2^ versus H/M) of Eu_8_CuNi_2.5_Si_42.5_ clathrate compound from the M(H) curves.

The isothermal magnetization with field M(H) at T = 50 K showed paramagnetic behavior at high magnetic fields. At intermediate temperature ranges (10 K ≤ T ≤ 30 K) the M(H) curves present weak ferromagnetic behavior at low magnetic fields (H ≤ 1 T). To identify the spontaneous magnetization, we analyzed the Arrot plot M^2^ versus H/M, as shown in Fig. 7. The low-temperature isothermal magnetization exhibited spontaneous magnetization at T = 2 and 5 K. The spontaneous magnetization at low temperatures is consistent with the magnetic hysteresis at T = 2 K in Fig. 6(a).

It is very interesting that spontaneous magnetization was observed in the regime of antiferromagnetic interaction, which strongly implies that the antiferromagnetic-like decrease in magnetic susceptibility near T = 15–18 K for ZFC χ(T) under H ≤ 0.1 T in Fig. 4(b) is not from stable AF ordering, but contains ferromagnetic clusters. This is also reasonable with the assumption of antiferromagnetic interaction between ferromagnetic clusters of Eu and Ni ions.

The conflicting phenomena of an AF-like decrease in χ(T) at low magnetic fields, and spontaneous magnetization at low-temperature, can occur for two reasons. One possibility is that the broad peaks in χ(T) at low magnetic field in Fig. 4(b) is a valence fluctuation. From the XPS measurements, we found a mixed valence state of Eu^2+^ and Eu^3+^ with respective proportions of 43 % and 57 %, in Fig. 3. Because Eu^3+^ is a non-magnetic ion, the valence fluctuation of Eu^2+^ and Eu^3+^ with decreasing temperature can appear as a broad peak in χ(T). However, this is not likely because under high magnetic fields the broad χ(T) peaks disappear and become ferromagnetic saturation, and there is significant thermal hysteresis between the ZFC and FC measurement process in Fig. 4(b).

The other possibility is the coexistence of AFM and FM ground states. The clathrate Eu_8_CuNi_2.5_Si_42.5_ contains two magnetic elements, Eu and Ni. The Eu^2+^ ion interacts by Rudermann-Kittel-Kasuya-Yoshida (RKKY) interaction while the Ni may have metallic ferromagnetism with Stoner's criterion. It is not surprising that the two different magnetic interactions may result in coexisting FM and AFM states. Therefore, it is believed that the ferromagnetic hysteresis at T = 2 K comes from the ferromagnetic cluster of Ni ions, and the decrease in χ(T) at low temperatures and low magnetic fields is caused by the multiple magnetic state of FM to AFM with decreasing temperatures.

### 3.4 Electrical transport and magnetocaloric properties

**Fig. 8:**
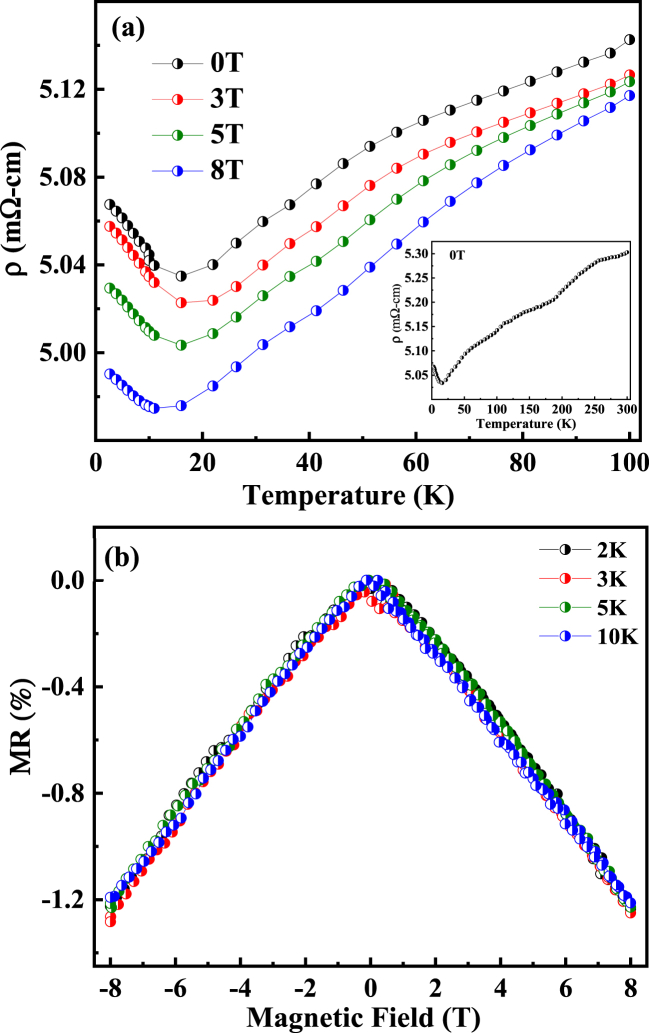
(a) Temperature-dependent electrical resistivity ρ(T) of the Eu_8_CuNi_2.5_Si_42.5_ clathrate under various magnetic fields, as indicated. (b) Isothermal magnetoresistance [ρ(H)- ρ(0)]/ρ(0)ⅹ100 (%) versus magnetic field ranging from -8T < H < 8T at different temperatures, as indicated. The full measurement of electrical resistivity vs temperature is shown in the inset of Fig. 8 (a).

The temperature-dependent electrical resistivity measurements ρ(T) of the Eu_8_CuNi_2.5_Si_42.5_ clathrate under static magnetic fields are shown in Fig. 8(a). There is no distinctive magnetic ordering feature near 30 K, but there is a clear increase in resistivity below T < 15 K. The increase in ρ(T) at low temperature is associated with the AFM state below T < 15 K. In general, Hund coupling gives rise to spin-dependent electrical transport, such as the FM-metal and AFM-insulating phases. Under ferromagnetic parallel spin alignment, the hopping of spin-polarized electrons to the nearest neighbor maximizes spin angular momentum. On the other hand, the spin polarized electronic hopping under the antiferromagnetic background decreases the spin angular momentum, which violates Hund's rule, resulting in an increase in electrical resistivity. Therefore, the increase in ρ(T) at low temperature also supports the conclusion of a multiple magnetic ground state from FM to AFM spin alignment.

When we apply magnetic fields, the spin reorientation decreases electrical resistivity, giving rise to negative magnetoresistance. The isothermal magnetoresistances [ρ(H)-ρ(o)/ρ(0)] with applied magnetic field at different temperatures are presented in Fig. 8(b). It shows linear negative magnetoresistance. Such negative MR behavior is widely observed in FM materials. The spin reorientation with increasing magnetic field can decrease the electrical resistivity due to the Hund coupling of spin dependent electronic transport. However, the linear MR with magnetic field is the result of the quantum limit of electrical transport. For a gapless semiconductor, the quantum limit of the Landau band can lead to linear quantum magnetoresistance [31]. Various gapless semiconductors have already been shown to exhibit such a quantum linear MR in very small transverse magnetic fields [32]. Large and linear non-saturating MR was observed in a linear band dispersion with two-charge carrier behavior at low temperatures [33].

**Fig. 9:**
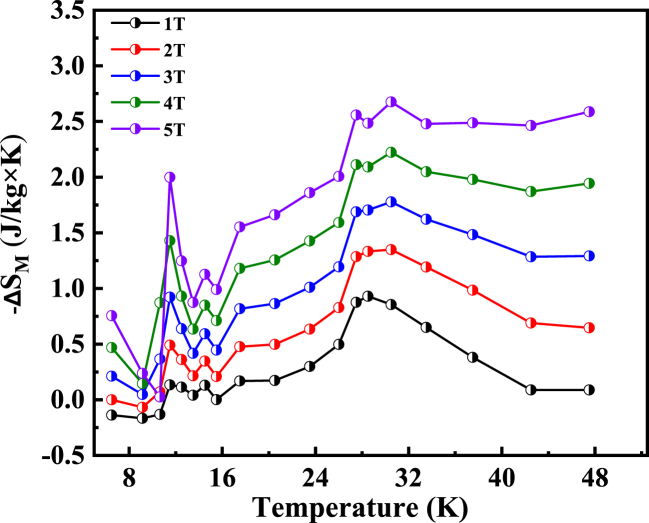
Temperature-dependent magnetic entropy change $\Delta S_{M}$ of the Eu_8_CuNi_2.5_Si_42.5_ clathrate under various magnetic fields, as indicated.

The magnetocaloric properties of the compound were obtained from the M(H) curves using Maxwell relation ΔS_M_ = μ_0_ ∫(dM/dT)_H_dH. The variation in magnetic entropy with temperature under different magnetic fields is shown as Fig. 9. It was found that the magnetic entropy change (-ΔS_M_) is closely related to the phase transition temperature, for both FM and AFM. The two maximum values of entropy (-ΔS_M_) were observed for AFM and FM transitions. The entropy change reached to be 2.02 J kg^−1^ K^−1^ and 2.71 kg^−1^ K^−1^ for 5T respectively, and is quite comparable with the earlier reported data for a Eu containing clathrate [13]. Most of the MCE showed the peak was near the magnetic phase transition [34]. On a magnetic entropy graph, it's conceivable to detect extra peaks at lower temperatures, particularly in materials displaying intricate magnetic characteristics. These lower-temperature peaks may be linked to phenomena such as shifts in magnetic phases, the alignment of magnetic moments, or the initiation of magnetic order within the material.

On the other hand, it is noteworthy that the Eu_8_CuNi_2.5_Si_42.5_ clathrate showed high MCE properties around its magnetic transition temperature range. The concept of a table-like Magnetocaloric Effect (MCE) has been observed in certain magnetic materials that undergo multiple successive magnetic transitions or exhibit magnetic field-sensitive behaviors [34]. This behavior has also been observed in some composite materials. Consequently, a key objective is to identify or engineer Magnetocaloric materials with a well-defined MCE pattern over a broad temperature range, which is crucial for practical applications. This can be indirectly assessed by examining the refrigerant capacity (RC) parameter. To assess the performance of Eu_8_CuNi_2.5_Si_42.5_ clathrate, we've conducted calculations for refrigerant capacity (RC), a crucial parameter in evaluating and approving cooling efficiency. A significant entropy change across a wide temperature range indicates a substantial refrigerant capacity [34].

$$RC=-\int_{T_{1}}^{T_{2}}\Delta S_{M}\left(T\right)dT$$

Refrigerant capacity (RC) is not solely determined by the maximum of -ΔS_M_(T) but also by the entire profile of -ΔS_M_(T). RC is calculated through numerical integration of the area under the -ΔS_M_(T) curve. The temperature integration boundaries are defined by the half-maximum of the ΔS_M_(T) peak, with T_1_ and T_2_ representing the temperatures at which the peak reaches half its maximum value, serving as the limits for integration [34,35]. The RC value for Eu_8_CuNi_2.5_Si_42.5_ clathrate calculated to be 78 J/kg at 5T magnetic field, an exceptionally large value for low temperature magnetocaloric materials [36].

## 4 Conclusions

In summary, we synthesized the Eu_8_CuNi_2.5_Si_42.5_ clathrate compound by arc melting with an annealing method. A mixed valance state of Eu^2+^ and Eu^3+^ was observed in the X-ray photoemission spectroscopy measurements. Magnetic measurements in low field and low-temperature range revealed multiple magnetic ordering, from ferromagnetic near 35 K to antiferromagnetic ordering (below 25 K). There was a spin-reorientation regime from the field- and temperature-dependent magnetization. With increasing magnetic field, there was a broadening of the AFM peaks and finally a transition to the FM state under high magnetic fields due to spin reorientation. The magnetic reentrance from the FM to AFM state was also confirmed in the specific heat measurement. The magnetic hysteresis and Arrot plot analyses showed spontaneous magnetization at low temperatures. The spontaneous magnetization in the AFM state at low temperatures indicates the coexistence of a magnetic ground state with AFM ordering by Eu^2+^ ions, and ferromagnetic clusters of Ni^2+^ ions, respectively. The magnetocaloric analyses of the Eu_8_CuNi_2.5_Si_42.5_ compounds revealed high spin entropy change over a wide temperature range, indicating high refrigerant capacity from low-temperature magnetocaloric materials.

## CRediT authorship contribution statement

**Pooja Rawat:** Writing – original draft, Visualization, Validation, Methodology, Investigation, Funding acquisition, Formal analysis, Data curation. **Seung Hun Cha:** Methodology, Investigation, Formal analysis, Data curation. **Jin Hee Kim:** Investigation, Formal analysis, Data curation. **Jae Hyun Yun:** Methodology, Formal analysis, Data curation. **Jong-Soo Rhyee:** Writing – review & editing, Supervision, Resources, Project administration, Investigation, Funding acquisition, Formal analysis, Data curation, Conceptualization.

## Declaration of competing interest

The authors declare that they have no known competing financial interests or personal relationships that could have appeared to influence the work reported in this paper.
